# First Characterization and Regulatory Function of piRNAs in the *Apis mellifera* Larval Response to *Ascosphaera apis* Invasion

**DOI:** 10.3390/ijms242216358

**Published:** 2023-11-15

**Authors:** Minghui Sun, Xiaoxue Fan, Qi Long, He Zang, Yiqiong Zhang, Xiaoyu Liu, Peilin Feng, Yuxuan Song, Kunze Li, Ying Wu, Haibin Jiang, Dafu Chen, Rui Guo

**Affiliations:** 1 College of Animal Sciences (College of Bee Science), Fujian Agriculture and Forestry University, Fuzhou 350002, China; hnmhsun@126.com (M.S.); imfanxx@163.com (X.F.); l1070190695@126.com (Q.L.); zanghe321@163.com (H.Z.); zhangyiqiong1121@163.com (Y.Z.); liuxiaoyu2000@163.com (X.L.); fengwangman@163.com (P.F.); syx_wyy@outlook.com (Y.S.); kunze0515@163.com (K.L.); dfchen826@fafu.edu.cn (D.C.); 2 Apiculture Science Institute of Jilin Province, Jilin 132000, China; wy569703@163.com (Y.W.); jhb18047513706@163.com (H.J.); 3 National & Local United Engineering Laboratory of Natural Biotoxin, Fuzhou 350002, China; 4 Apitherapy Research Institute of Fujian Province, Fuzhou 350002, China

**Keywords:** honeybee, *Apis mellifera ligustica*, chalkbrood, *Ascosphaera apis*, piRNA, regulation

## Abstract

piRNAs are a class of small non-coding RNAs that play essential roles in modulating gene expression and abundant biological processes. To decode the piRNA-regulated larval response of western honeybees (*Apis mellifera*) to *Ascosphaera apis* infection, the expression pattern of piRNAs in *Apis mellifera ligustica* larval guts after *A. apis* inoculation was analyzed based on previously obtained high-quality small RNA-seq datasets, followed by structural characterization, target prediction, regulatory network investigation, and functional dissection. Here, 504, 657, and 587 piRNAs were respectively identified in the 4-, 5-, and 6-day-old larval guts after inoculation with *A. apis*, with 411 ones shared. These piRNAs shared a similar length distribution and first base bias with mammal piRNAs. Additionally, 96, 103, and 143 DEpiRNAs were detected in the 4-, 5-, and 6-day-old comparison groups. Targets of the DEpiRNAs were engaged in diverse pathways such as the phosphatidylinositol signaling system, inositol phosphate metabolism, and Wnt signaling pathway. These targets were involved in three energy metabolism-related pathways, eight development-associated signaling pathways, and seven immune-relevant pathways such as the Jak-STAT signaling pathway. The expression trends of five randomly selected DEpiRNAs were verified using a combination of RT-PCR and RT-qPCR. The effective overexpression and knockdown of piR-ame-945760 in *A. apis*-infected larval guts were achieved by feeding a specific mimic and inhibitor. Furthermore, piR-ame-945760 negatively regulated the expression of two target immune mRNAs, *SOCS*5 and *ARF*1, in the larval gut during the *A. apis* infection. These findings indicated that the overall expression level of piRNAs was increased and the expression pattern of piRNAs in larval guts was altered due to the *A. apis* infection, DEpiRNAs were putative regulators in the *A. apis*-response of *A. m. ligustica* worker larvae. Our data provide not only a platform for the functional investigation of piRNAs in honeybees, especially in bee larvae, but also a foundation for illuminating the piRNA-involved mechanisms underlying the host response to the *A. apis* infection.

## 1. Introduction

The western honeybee (*Apis mellifera*) is widely reared in China and many other countries and provides pollination services for a substantial quantity of wild flowers and agricultural crops, thus playing crucial economic and ecological roles. However, as a eusocial insect, *A. mellifera* is susceptible to infections by various pathogens and parasites. Among these, *Ascosphaera apis* is an obligated lethal fungal pathogen that exclusively infects bee larvae and causes chalkbrood disease, which results in a dramatic reduction in colony population and productivity [1].

Non-coding RNAs (ncRNAs) are diverse and pivotal regulators in gene expression and biological processes, from cell proliferation, differentiation, apoptosis, and autophagy to growth, development, metabolism, immune defense, and host–pathogen interaction [2]. piRNAs (Piwi-interacting RNAs) are a class of small ncRNAs with a length distribution ranging from 23 nt to 35 nt [3,4]. piRNA was first discovered in *Drosophila* and then in mice and *Caenorhabditis elegans* [5,6]. Thereafter, increasing numbers of piRNAs were identified in insects such as *Drosophila* [7], *Bombyx mori* [8], and *Aedes aegypti* [9]. A major function of piRNAs is to specifically bind to Piwi family proteins, as well as members of the Piwi, Aub (Aubergine), and Ago3 (Argonaute3), further silencing the activities of transposons [10]. Furthermore, piRNAs have been demonstrated to be involved in an array of life activities such as genome rearrangement and epigenetic regulation and to serve as novel biomarkers and therapeutic targets for disease [11,12]. In insects, piRNAs are suggested to participate in the modulation of cognate viral infection, metabolic homeostasis, gut development, and sex determination [13,14,15,16]. Moreover, accumulating evidence has shown that piRNAs are crucial regulators in responses of insects to pathogen or parasite invasion. For instance, Feng et al. [17] found that the differential expression of piRNAs in the fat body was significantly higher than in the midgut during BmNPV (Bombyx mori nucleopolyhedrovirus) infection and provided information on the interaction between DEpiRNAs and their putative targets, which may be important during BmNPV infection. However, studies on the regulatory functions of piRNAs in interactions between honeybees and pathogens or parasites have been lacking up to now. Our previous studies indicated that various types of ncRNAs such as miRNAs and circRNAs are engaged in the responses of *A. mellifea* larvae to *A. apis* invasion [18,19]. In view of the involvement of piRNAs in the immune responses of insects [17], we raise the question of whether piRNAs participate in modulating the responses of *A. mellifera* larvae to *A. apis* infection.

In our previous work, high-quality transcriptome datasets from the small RNA sequencing (sRNA-seq) of *A. apis*-inoculated and un-inoculated *Apis mellifera ligustica* larval guts were obtained. Here, piRNAs in the gut tissues of *A. m. ligustica* larvae inoculated with *A. apis* were characterized, followed by an investigation of the differential expression pattern of piRNA (DEpiRNA) in the host response to fungal infection, as well as target prediction and analysis. The potential regulatory function of DEpiRNAs in larval responses, especially the immune response, was further resolved. To the best of our knowledge, this is the first report of a piRNA-mediated bee larval response to *A. apis* infection. Our findings not only offer new insight into the interaction between *A. mellifera* larvae and *A. apis* but also lay the groundwork for clarifying the mechanism underlying the piRNA-mediated host response.

## 2. Results

### 2.1. Number, Characteristics, and Overall Expression Level of piRNAs in A. m. ligustica Larval Guts Inoculated with A. apis

**Figure 1:**
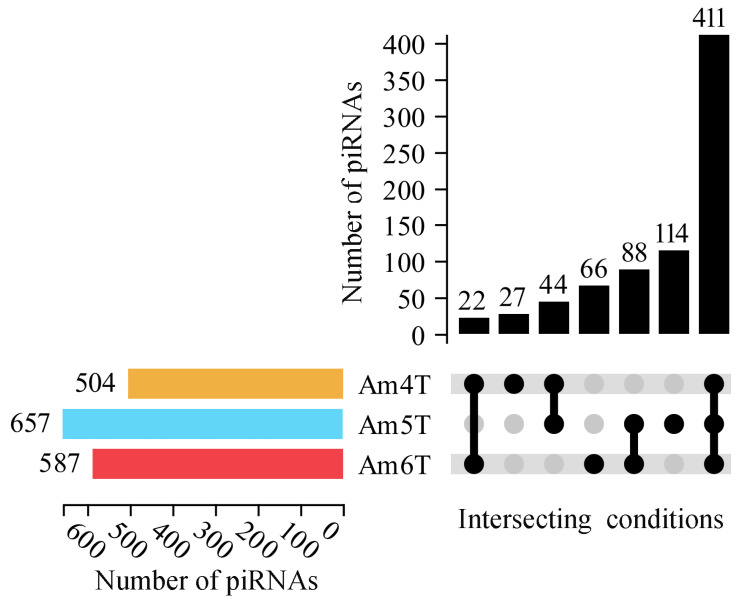
UpSet plot of total piRNAs and shared piRNAs by *A. m. ligustica* worker 4-, 5-, and 6-day-old larval guts infected by *A. apis*. The histogram graph at the lower left indicates total piRNAs identified in the Am4T, Am5T, and Am6T groups. The UpSet diagram at the lower right indicates piRNAs shared by two or three groups or those unique for one group the single black node represents piRNAs belonging to corresponding group, two black nodes connected by a black line represents there are piRNAs shared by corresponding two groups, the grey node represents none piRNAs distributed in a certain leghth in corresponding group; and the number of unique and common piRNA are presented above; while the histogram graph at the upper right indicates the numbers of corresponding shared or unique piRNAs.

Here, 504, 657, and 587 piRNAs were discovered in the *A. apis*-inoculated *A. m. ligustica* worker 4-, 5-, and 6-day-old larval gut tissues (named Am4T, Am5T, and Am6T groups), respectively (Figure 1). After removing redundant ones, a total of 772 *A. m. ligustica* piRNAs were identified. Additionally, 411 piRNAs were shared by the three groups, while the numbers of unique ones were 27, 66, and 114, respectively (Figure 1).

**Figure 2:**
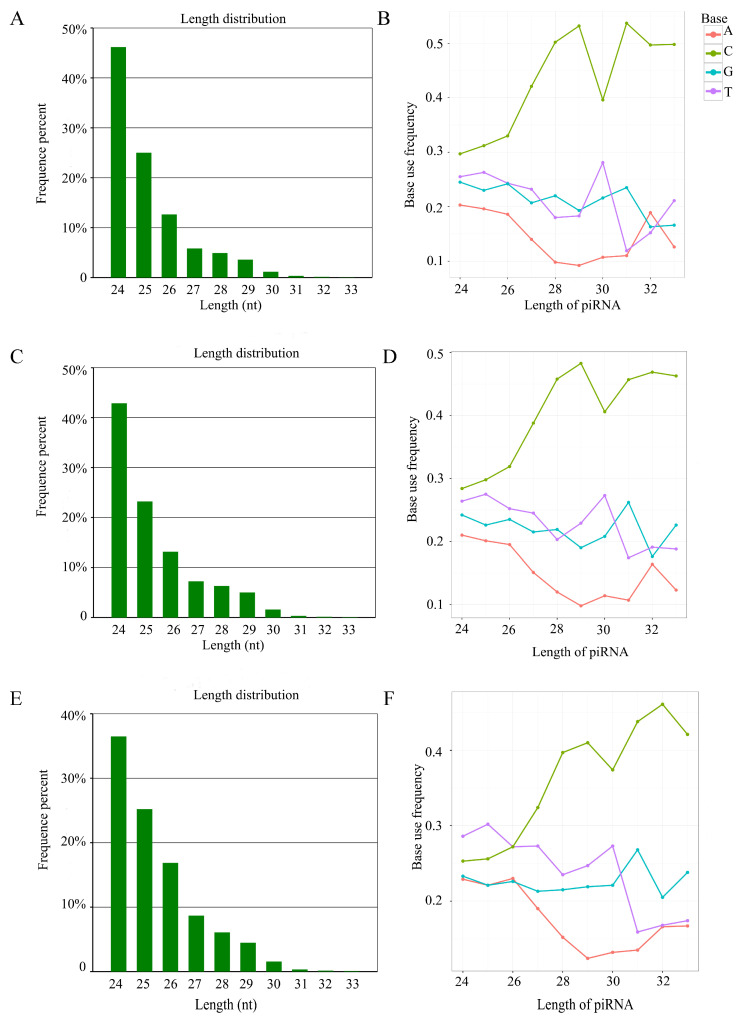
Length distribution and first base bias of the identified piRNAs in *A. apis*-inoculated larval guts. (**A**,**B**) Length distribution and first base bias of piRNAs in the Am4T group; (**C**,**D**) Length distribution and first base bias of piRNAs in Am5T group; (**E**,**F**) Length distribution and first base bias of piRNAs in the Am6T group.

Additionally, the length distribution of the identified piRNAs in *A. apis*-inoculated larval guts ranged from 24 nt to 33 nt (Figure 2A,C,E). Furthermore, the first base of these piRNAs had a “C” bias (Figure 2B,D,F).

### 2.2. Differential Expression Pattern of piRNAs in A. m. ligustica Larval Guts Following A. apis Infection

**Figure 3:**
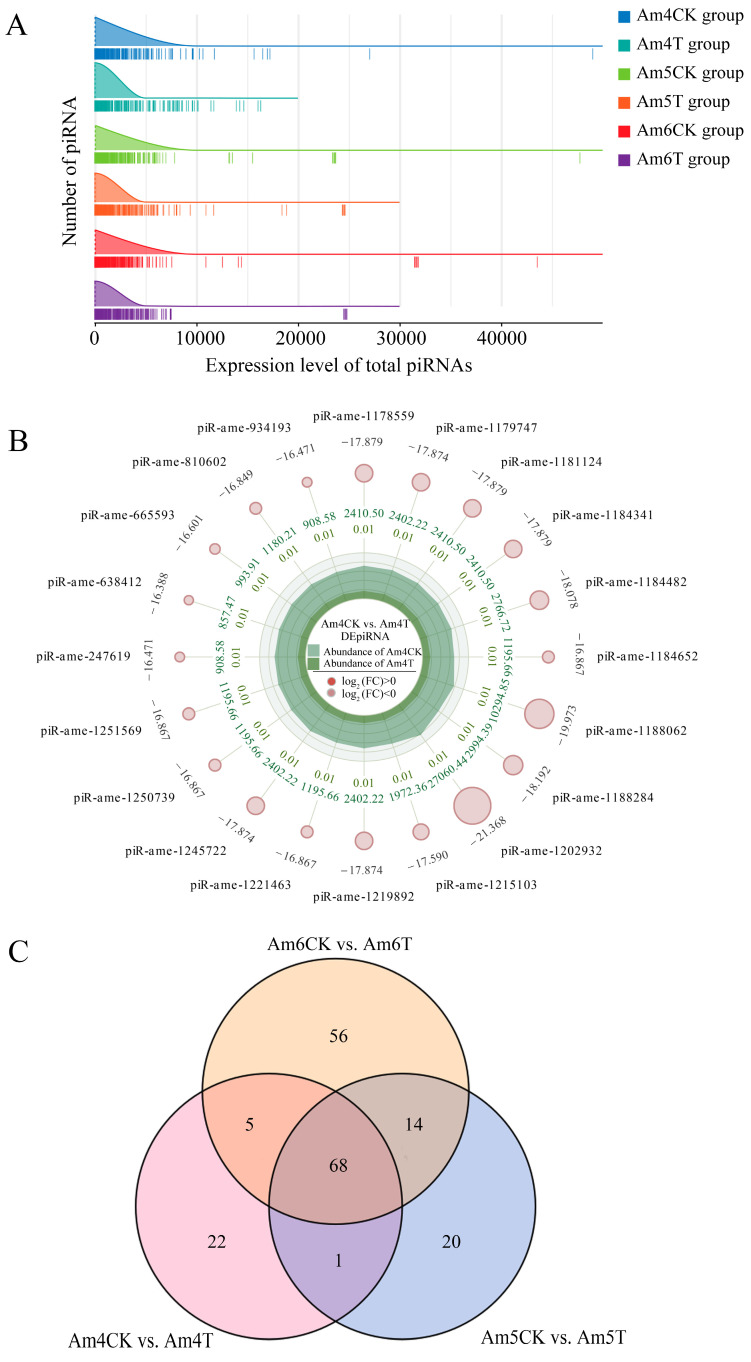
Overall expression level and differential expression profile of piRNAs in *A. apis*-inoculated and uninoculated *A. m. ligustica* worker larval guts. (**A**) Ridgeline plot of the expression level of the total piRNAs in six groups; the peak of the ridge indicates the most abundant piRNA in a certain group, and each vertical line indicates a piRNA. (**B**) Radar map of the top 20 DEpiRNAs in the Am4CK vs. Am4T comparison group; green circles indicate down-regulated piRNAs, and the size of each circle indicates the numerical value of log_2_(FC); data outside the circles indicate the average expression of piRNAs in the Am4CK group, and inner-circle data represent the average expression of sample Am4T; the light and deep green colors at the center repectively indicate the expression abundance of Am4CK and Am4T on each axis. (**C**) Venn diagram of DEpiRNAs discovered in the three comparison groups.

Further investigation suggested that the overall expression levels of piRNAs among three *A. apis*-inoculated or uninoculated groups were similar, whereas the overall expression level of piRNAs in the *A. apis*-inoculated larval gut was higher than in the uninoculated larval gut (Figure 3A). In the Am4CK vs. Am4T comparison group, 8 up-regulated piRNAs and 88 down-regulated ones were identified, as shown in Figure 3B (see also Table S3). In the Am5CK vs. Am5T comparison group, 26 up-regulated and 77 down-regulated piRNAs were detected (Figure S1A, see also Table S3), whereas 61 up-regulated and 82 down-regulated ones were discovered in the Am6CK vs. Am6T comparison group (Figure S1B, see also Table S3). Moreover, the Venn analysis showed that a total of 68 piRNAs were shared by these three comparison groups; the numbers of unique ones were 22, 20, and 56, respectively (Figure 3C).

### 2.3. Annotation and Analysis of DEpiRNA-Targeted mRNAs

**Figure 4:**
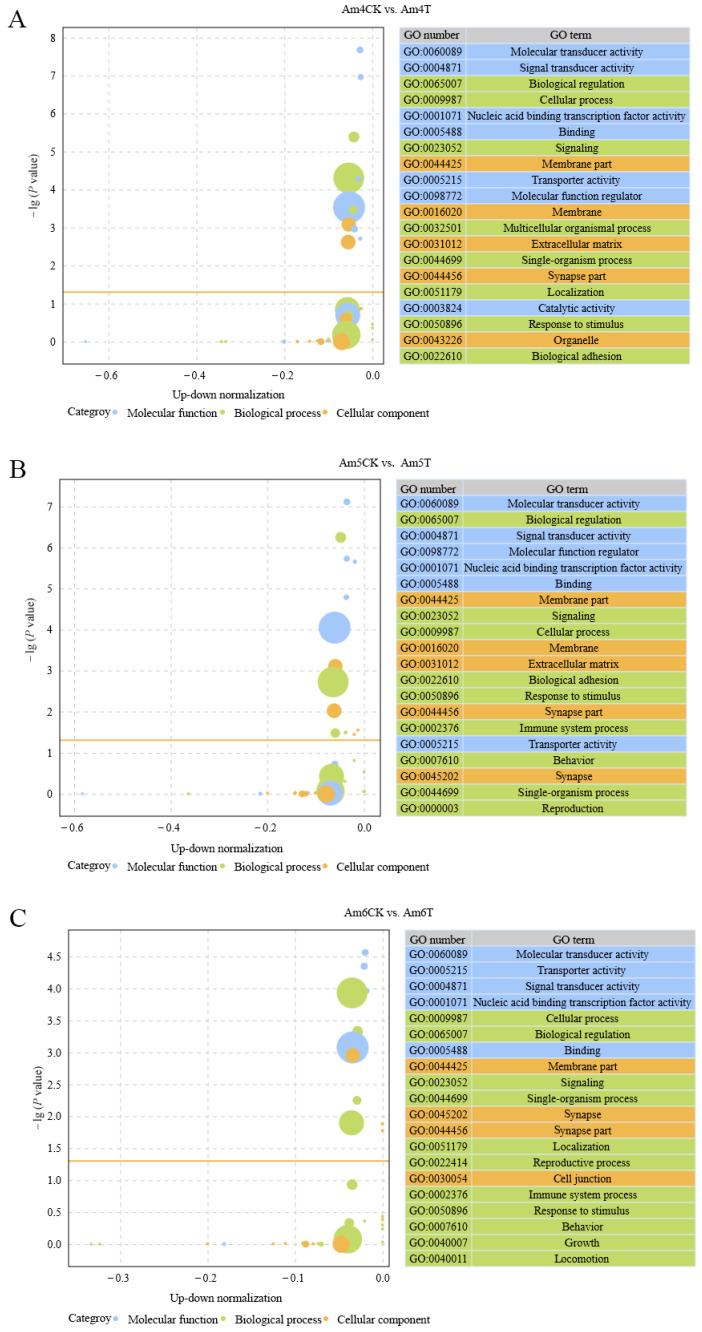
Bubble diagrams of the top 20 GO terms annotated by the target mRNAs of DEpiRNAs in the Am4CK vs. Am4T (**A**), Am5CK vs. Am5T (**B**), and Am6CK vs. Am6T (**C**) comparison groups. The bubbles with three kinds of colors at the left represent various GO terms, the bubble size represents the number of target mRNAs, and the larger the circle size, the greater the number of target mRNAs; the orange line represents the threshold of *p* value = 0.05; the tables at the right present the number and name of the top 20 GO terms, and three kinds of colors represent three GO categories such as the molecular function, biological process, and cellular component, corresponding to the bubbles with different colors.

DEpiRNAs in the 4-day-old comparison group were predicted to target 11,517 mRNAs, which can be annotated to 48 GO terms, including 20 biological process-related terms such as biological regulation and cellular processes, 11 molecular function-related terms such as molecular transducer activity and signal transducer activity, and 17 cellular component-related terms such as the extracellular matrix and synapse part (Figure 4A). In contrast, the DEpiRNAs in the 5-day-old comparison group can target 11,255 mRNAs, which were involved in 20 biological process-associated terms such as biological regulation and signaling, 11 molecular function-associated terms such as molecular transducer activity and signal transducer activity, and 19 cellular component-associated terms such as the extracellular matrix and synapse part (Figure 4B). In addition, DEpiRNAs in the 6-day-old comparison group could target 12,077 mRNAs, which were engaged in 20, 11, and 17 terms related to the biological process, molecular function, and cellular component, respectively (Figure 4C).

**Figure 5:**
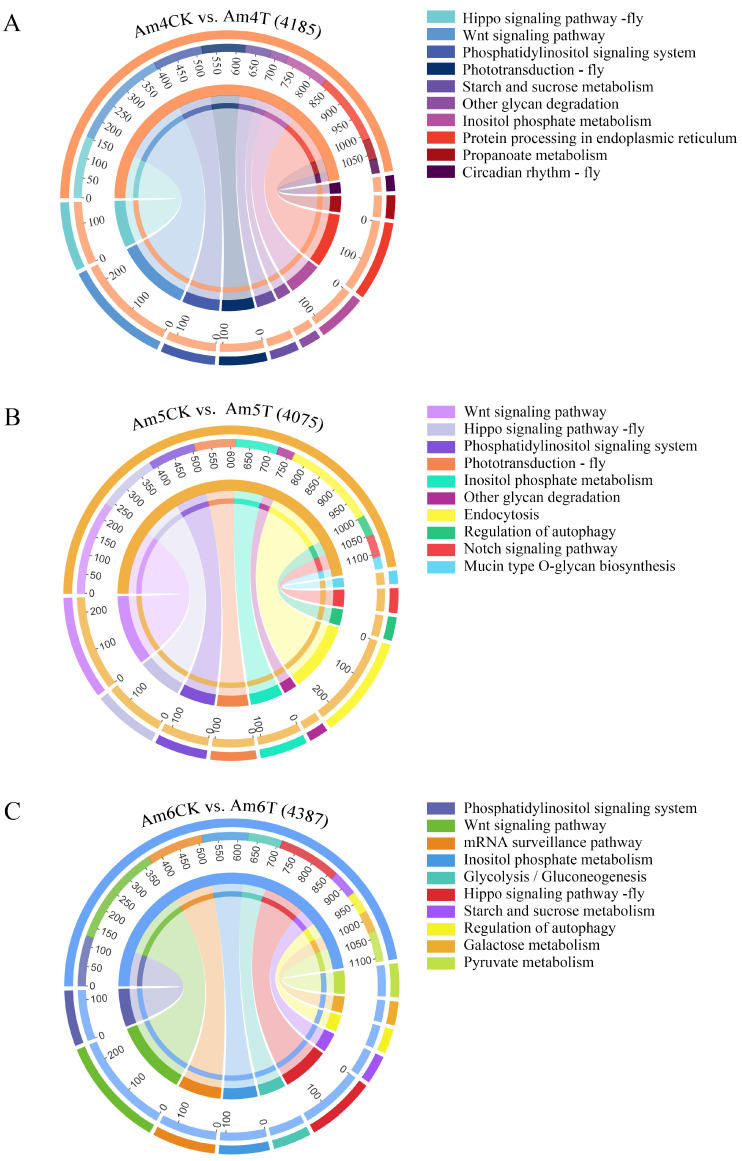
String diagrams of KEGG pathways annotated by the target mRNAs of DEpiRNAs in the Am4CK vs. Am4T (**A**), Am5CK vs. Am5T (**B**), and Am6CK vs. Am6T (**C**) comparison groups. Different colors represent different KEGG pathways. The scale value indicates the abundances of the source mRNAs annotated to the same KEGG pathway in all the source mRNAs targeted by DEpiRNAs in every comparison group.

DEpiRNAs in the 4-day-old comparison group were annotated to 151 KEGG pathways including the lysosome, the Notch signaling pathway, and oxidative phosphorylation (Figure 5A). Comparatively, DEpiRNAs in the 5-day-old comparison group were involved in 145 pathways including endocytosis, the Hippo signaling pathway, and nitrogen metabolism (Figure 5B). Additionally, DEpiRNAs in the 6-day-old comparison group were relevant to 151 KEGG pathways including the Jak-STAT signaling pathway*,* the Wnt signaling pathway, and sulfur metabolism (Figure 5C).

### 2.4. Regulatory Networks between DEpiRNAs and Target mRNAs

Complex regulatory networks were observed to be formed between DEpiRNAs and corresponding targets in the above-mentioned three comparison groups, and each DEpiRNA in the aforementioned three comparison groups had more than two targets (Table S4). Additionally, piR-ame-785504 and piR-ame-220719 in the 4-day-old comparison group were bound to the greatest number of targets (3020 and 2779), piR-ame-904316 and piR-ame-608008 in the 5-day-old comparison group targeted the most mRNAs (1919 and 1373), and piR-ame-854132 and piR-ame-806084 in the 6-day-old comparison group were found to link to the highest number of targets (2983 and 2700).

**Figure 6:**
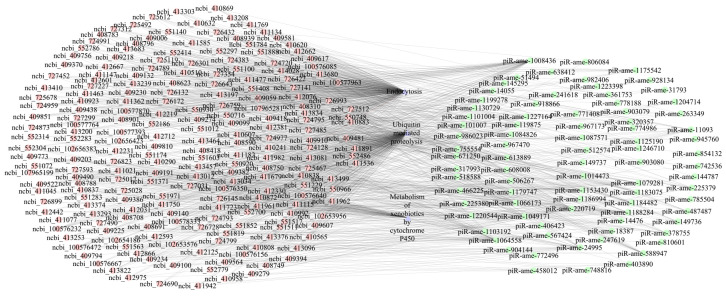
Immune pathway-associated regulatory network between DEpiRNAs and target mRNAs in the 4-, 5-, and 6-day-old comparison groups. Pink and green circles respectively indicate target mRNAs and DEpiRNAs, while purple triangles indicate immune pathways.

It is suggested that 300, 261, and 269 targets were involved in seven immune pathways, namely, ubiquitin-mediated proteolysis, the lysosome, the MAPK signaling pathway-fly, the metabolism of xenobiotics by cytochrome P450, drug metabolism–cytochrome P450, endocytosis, and the Jak-STAT signaling pathway (Figure 6; see also Table S4). In addition, 226, 226, and 229 target mRNAs in these three comparison groups were engaged in eight development-associated signaling pathways: Hippo, Wnt, FoxO, Notch, mTOR, TGF-beta, the hedgehog signaling pathway, and dorso-ventral axis formation (Figure S2A, Table S4), while 68, 61, and 85 targets were detected to be associated with three energy metabolism pathways: sulfur metabolism, nitrogen metabolism, and oxidative phosphorylation (Figure S2B, Table S4). Detailed information about the DEpiRNAs and corresponding targets is presented in Table S4.

### 2.5. RT-PCR and RT-qPCR Validation of DEpiRNA

**Figure 7:**
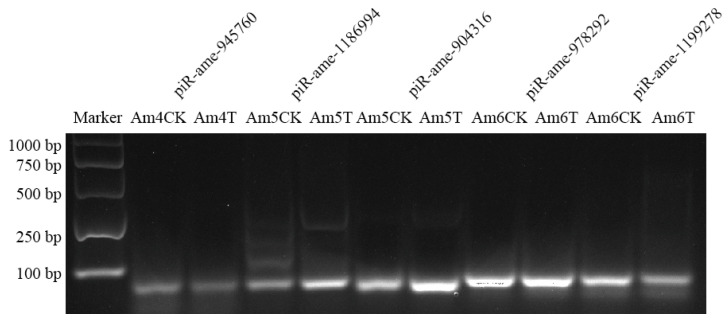
Agarose gel electrophoresis for the amplification of products from five DEpiRNAs.

RT-PCR results demonstrated that fragments with the expected size (about 70 bp) could be amplified from five randomly selected DEpiRNAs (Figure 7), confirming the expression of these DEpiRNAs in *A. m. ligustica* larval guts.

**Figure 8:**
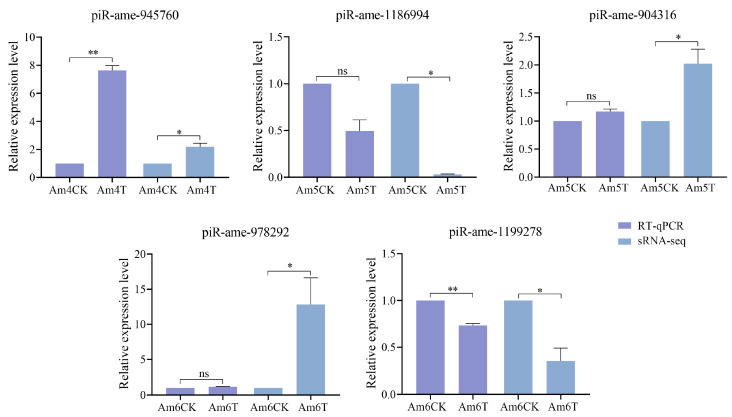
RT-qPCR detection of five DEpiRNAs. piR-ame-945760 was a DEpiRNA selected from the Am4CK vs. Am4T comparison group, piR-ame-1186994 and piR-ame-904316 were two DEpiRNAs selected from the Am5CK vs. Am5T comparison group, and piR-ame-978292 and piR-ame-1199278 were two DEpiRNAs selected from the Am6CK vs. Am6T comparison group. ns indicates non-significant, * indicates *p* < 0.05, and ** indicates *p* < 0.01.

In addition, the RT-qPCR results showed that the expression trends of these five DEpiRNAs were in accordance with those in the transcriptome data, confirming the reliability of the sRNA-seq datasets used in this work (Figure 8).

### 2.6. RT-qPCR Confirmation of Target mRNAs

**Figure 9:**
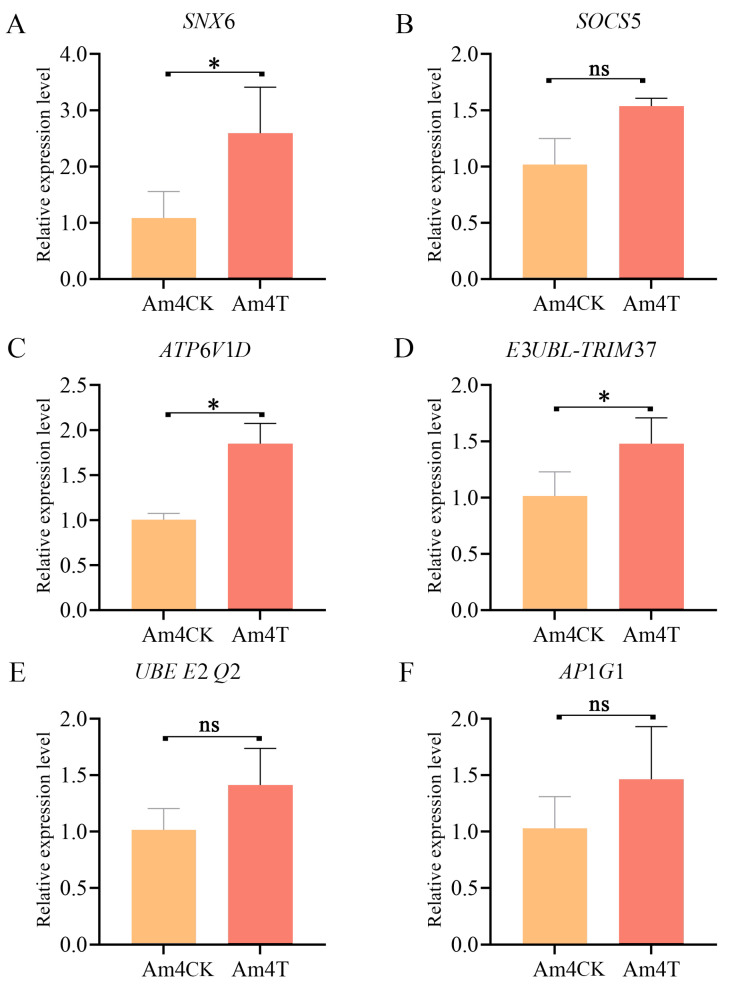
RT-qPCR determination of six target mRNAs of piR-ame-945760. These mRNAs were associated with endocytosis (**A**), the Jak-STAT signaling pathway (**B**), oxidative phosphorylation (**C**), ubiquitin-mediated proteolysis (**D**,**E**), and the lysosome (**F**). ns indicates non-significant, and * indicates *p* < 0.05.

Six immune-related mRNAs targeted by piR-ame-945760 were further chosen for RT-qPCR examination; the results demonstrated that the expression levels of these targets were all up-regulated in the *A. apis*-inoculated groups compared to those in the uninoculated groups, and the expression trends of these six targets and corresponding DEpiRNAs were similar (Figure 9).

### 2.7. Overexpression and Knockdown of piR-ame-945760 in A. apis-Infected Larval Guts

**Figure 10:**
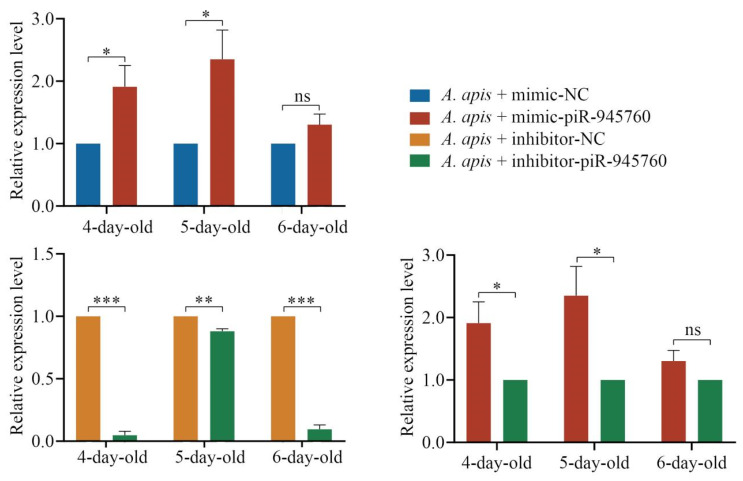
Detection of the relative expression level of piR-ame-945760 in *A. apis*-infected *A. m. ligustica* larval guts. Data were presented as the mean SD and subjected to Student’s *t*-test, ns indicates non-significant, * indicates *p* < 0.05, ** indicates *p* < 0.01, and *** indicates *p* < 0.001.

The RT-qPCR results suggested that, as compared with that in the *A. apis* + mimic-NC group, the expression level of piR-ame-945760 was significantly upregulated (*p* < 0.05) in the 4- and 5-day-old larval guts and upregulated (*p* > 0.05) in the 6-day-old larval gut in the *A. apis* + mimic-piR-945760 group (Figure 10). Additionally, the expression level of piR-ame-945760 in the guts of 4-, 5-, and 6-day-old larval guts in the *A. apis* + inhibitor-piR-945760 group was significantly downregulated (*p* < 0.01) in comparison with that in the *A. apis* + inhibitor-NC group (Figure 10). Moreover, as compared with that in the *A. apis* + inhibitor-piR-945760 group, the expression level of piR-ame-945760 was significantly upregulated (*p* < 0.05) in the 4- and 5-day-old larval guts and upregulated (*p* > 0.05) in the 6-day-old larval gut in the *A. apis* + mimic-piR-945760 group (Figure 10). These results demonstrated the effective overexpression and knockdown of piR-ame-945760 in the larval guts infected by *A. apis*.

### 2.8. Detection of the Relative Expression Level of SOCS5 and ARF1 in A. apis-Infected Larval Guts after the Overexpression and Knockdown of piR-ame-945760

**Figure 11:**
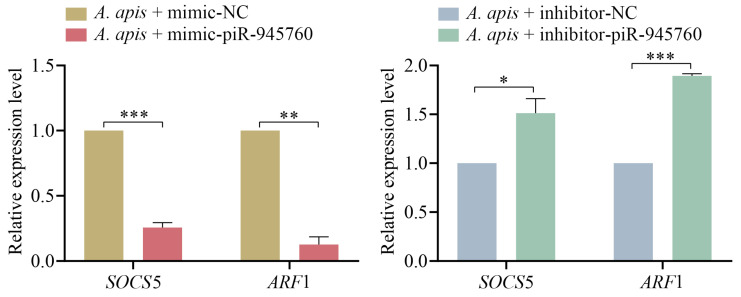
Determination of the relative expression levels of *SOCS*5 and *ARF*1 in the *A. apis*-infected *A. m. ligustica* 4-day-old larval guts after piR-ame-945760 overexpression and knockdown. Data were presented as the mean SD and subjected to Student’s *t*-test, ns indicates non-significant, * indicates *p* < 0.05, ** indicates *p* < 0.01, and *** indicates *p* < 0.001.

The RT-qPCR detection of two immune mRNAs was further conducted, and the results showed that the expression levels of both *SOCS*5 and *ARF*1 were significantly downregulated (*p* < 0.01) in the 4-day-old larval gut in the *A. apis* + mimic-piR-945760 group in comparison with those in the *A. apis* + mimic-NC group (Figure 11); additionally, as compared with those in the *A. apis* + inhibitor-NC group, the expression levels of both *SOCS*5 and *ARF*1 were significantly upregulated (*p* < 0.05) in the *A. apis* + inhibitor-piR-945760 group (Figure 11).

## 3. Discussion

Though piRNAs have been reported to be involved in interactions between mammals [20,21] or insects [22] and pathogens, little was known about the piRNA-engaged responses of honeybees to pathogen infections. Here, based on the obtained high-quality sRNA-seq datasets, 504, 657, and 587 piRNAs were discovered for the first time in the gut tissues of *A. m. ligustica* worker 4-, 5-, and 6-day-old larvae challenged by *A. apis*. In addition, 411 piRNAs were shared by the Am4T, Am5T, and Am6T groups (Figure 1), suggesting that these shared piRNAs may play vital roles during the *A. apis* infection process. We previously identified 775, 831, and 765 piRNAs in the un-inoculated 4-, 5-, and 6-day-old larval guts [16]. It was observed that 592 (62.45%) piRNAs were shared by the *A. apis*-inoculated and uninoculated larval guts, indicative of the fundamental roles of these shared piRNAs in both the development and the fungal response of larval guts. Combining the piRNAs identified in the *A. apis*-inoculated and uninoculated larval guts, a total of 948 non-redundant piRNAs were found. Given that the quantity of known piRNAs in honeybees at present is very limited, these piRNAs could enrich the reservoir of *A. mellifera* piRNAs and provide a valuable resource for related studies. Piwi proteins preferentially stabilize piRNA precursor intermediate fragments with a base, thus generating a nucleotide bias that is inherited by the mature piRNA [23]. It was documented that phased piRNAs beginning with “U” could be produced by a processive nuclease complex measuring out ~26 nt and then cleaving at the nearest “U” [24,25]. Alternatively, they could be made by the same nuclease measuring out ~26 nt but cleaving at all nucleotides with a similar efficiency; the subsequent binding of Piwi and Aub would select for piRNAs starting with “U”. Here, the identified piRNAs in the *A. apis*-inoculated larval guts ranged from 26 nt to 33 nt and had a “C” bias, analogous to the properties of the piRNAs identified in uninoculated *A. m. ligustica* larval guts and some other mammals such as mice [26]. The results demonstrated that the characteristics of *A. m. ligustica* piRNAs were unchanged under *A. apis* infection, reflecting the structural stability of piRNAs, as reported in other species [27].

Increasing evidence has shown that piRNAs are involved in the responses of insects to pathogen infections. For example, Morazzani et al. [28] discovered that arbovirus replication in *Mosquito soma* was capable of triggering the host piRNA pathway. The silencing of piRNA-associated proteins reduced virus-specific piRNA-like molecules and enhanced viral replication and production. Also, the piRNA pathway was detected to exert an anti-RNA virus function in cultured cells of lepidopteron like silkworm (*Bombycis mori*) [29]. Previously, we found that the expression pattern of piRNAs in the *A. m. ligustica* workers’ midguts was altered due to infection by *Nosema ceranae*, another prevalent bee fungal parasite [30]. Here, we observed that the overall expression level of piRNAs in the *A. apis*-inoculated larval guts was higher than in the uninoculated larval guts, indicating the impact of *A. apis* infection on piRNA expression at an integral level. Additionally, 96, 103, and 143 piRNAs were differentially expressed in the 4-, 5-, and 6-day-old larval guts after *A. apis* infection, further suggesting that the *A. apis* infection changed the expression profile of piRNAs in the larval guts. Moreover, the number of DEpiRNAs grew with the infection time, implying that more piRNAs were employed by the host in response to the fungal invasion. It is inferred that this may be a strategy of the host to respond to the *A. apis* infection. Intriguingly, a portion of piRNAs, such as piR-ame-149736 and piR-ame-1202932 in the 4-day-old larval gut, piR-ame-1066173 and piR-ame-1202932 in the 5-day-old larval gut, and piR-ame-1125190 and piR-ame-1202932 in the 6-day-old larval gut, were observed to be highly up- or down-regulated during the larval *A. apis*-response, deserving additional work to decipher their regulatory functions.

In recent years, studies have demonstrated that piRNAs are pivotal regulators in gene expression and diverse life activities, in a similar way to miRNAs [31,32]. In this study, DEpiRNAs in the 4-, 5-, and 6-day-old comparison groups were predicted to target 11,517, 11,255, and 12,077 mRNAs, respectively. These targets are engaged in a series of critical functions such as molecular transducer activity, biological regulation, and membranes, as well as an array of pathways of importance including five cellular and two humoral immune pathways, e.g., 83, 87, and 123 DEpiRNAs in 4-, 5, and 6-day-old larval guts infected by *A. apis* potentially targeted 109, 107, and 108 mRNAs relative to endocytosis, while 78, 89, and 122 DEpiRNAs had 83, 80, and 86 target mRNAs associated with ubiquitin-mediated proteolysis. Endocytosis and phagocytosis are two major cellular immune pathways in the honeybee [1]. The ubiquitin–proteasome system plays a vital part in stress response, host adaptation, and fungal pathogenesis [33]. Altogether, these results were suggestive of an extensive regulatory role of DEpiRNAs in the larval response to *A. apis* invasion, including a cellular immune response, which was in accordance with associated documentations of piRNAs in other species, including humans and insects [17,34].

Multiple immune signaling pathways, including Toll, IMD, JAK/STAT, JNK, and insulin, are engaged in insect immunity [35]. The JAK/STAT signaling pathway is a universally expressed intracellular signal transduction pathway involved in many important biological processes, including cell proliferation, differentiation, apoptosis, and immune regulation, and has been shown to be involved in antimicrobial, antiviral, and antimalarial responses; it is the main signaling mechanism of many cytokines and growth factors [35,36]. MAP kinase is one of the oldest and most evolutionarily conserved signaling pathways and is critical for many immune processes, including innate immunity, adaptive immunity, and the initiation of immune responses to activation-induced cell death [37]. Here, 83, 87, and 122 DEpiRNAs in the 4-, 5-, and 6-day-old comparison groups were detected to target 70, 69, and 69 mRNAs, which were engaged in humoral immune pathways such as MAPK and the Jak-STAT signaling pathway, suggesting the involvement of corresponding DEpiRNAs in the larval humoral immune response (Figure 6). In summary, these results uncovered that DEpiRNAs are putative modulators in both the cellular and humoral immune responses of *A. m. ligustica* larval guts to *A. apis* infection.

Previously, the overexpression and knockdown of piRNAs were reported in studies relevant to mammals and insects such as humans and *Culex pipiens pallens* [38,39]. For instance, through the overexpression and knockdown of piRNA-3312, Guo et al. [39] found that piRNA-3312 targets the gut esterase 1 gene to negatively regulate resistance to the insecticide deltamethrin. In this work, a specific mimic and inhibitor for piR-ame-945760 were designed and used to feed larvae of *A. m. ligustica* workers, and the RT-qPCR results were indicative of the effective overexpression and knockdown of piR-ame-945760 in the larval guts infected by *A. apis* (Figure 10), which verified the feasibility of the functional investigation of honeybee piRNAs via the feeding method. This is the first documentation of the overexpression and knockdown of bee piRNA, offering a solid basis for the further functional study on piRNAs in honeybees, especially in bee larvae.

Suppressor of cytokine signaling (SOCS) proteins were identified as the inducible feedback inhibitors of various hematopoietic cytokines triggering the JAK/STAT signaling pathway, playing an important role in inhibiting immunity and inflammation [40,41]. ADP-ribosylation factor 1 (*ARF*1) is recognized to largely regulate the immune responses of host cells in *Haemonchus contortus* [42]. The transporter ADP ribosylation factor 1(*ARF*1) differentially regulated humoral immunity in *Drosophila* and played a pivotal part in regulating the cellular immune response by controlling the crystal cell melanization and phenoloxidase activity with the blood cell-specific endosomal regulator *ASRIJ* [43]. Here, *SOCS*5 *and ARF*1, two crucial immune mRNAs targeted by piR-ame-945760, were found to be significantly downregulated in the 4-day-old larval gut infected by *A. apis* after the overexpression of piR-ame-945760 (Figure 11), whereas their significant upregulation was observed in the *A. apis*-infected 4-day-old larval gut after piR-ame-945760 knockdown (Figure 11). The results confirmed the negative regulation relationship between the piR-ame-945760 and target mRNAs of the aforementioned two immune genes. Hence, piR-ame-945760 was suggested to participate in the regulation of the host response to *A. apis* infection through the negative modulation of *SOCS*5 and *ARF*1 expression. More efforts are required to elucidate the mechanism underlying the larval response to the *A. apis* challenge mediated by the piR-ame-945760-*SOCS*5/*ARF*1 axis in the near future.

NcRNAs have been reported to be involved in the immune response of honeybees against fungal invasion [19,44]. The role of piRNAs in the immune response of insects has been documented, particularly in their antiviral defense against RNA viruses [4,45]. However, their universality in this regard remains a subject of debate. In this context, we, for the first time, investigated the expression pattern and regulatory function of piRNAs in the larval guts of *A. m. ligustica* workers during *A apis* infection. Our findings provided compelling evidence for a previously unrecognized role of piRNAs in the host response to fungal invasion by characterizing the dynamic changes in DEpiRNAs during *A. apis* infection and highlighted the potential utility of piRNAs as targets for therapeutic interventions in honeybees and other insects.

## 4. Materials and Methods

### 4.1. Bee Larvae and Fungi

*A. m. ligustica* larvae were gained from three colonies reared in the apiary of the College of Animal Sciences (College of Bee Science), Fujian Agriculture and Forestry University, Fuzhou City, China. *A. apis* was previously isolated from a fresh chalkbrood mummy of *A. m. ligustica* larvae [46] and conserved in our laboratory and the China General Microbiological Culture Collection Center (CGMCC) under the microbiological culture collection number: 40,895.

### 4.2. sRNA-Seq Data Source

In a previous study, *A. apis*-inoculated 4-, 5-, and 6-day old larval gut samples (Am4T, Am5T, and Am6T groups), and corresponding uninoculated 4-, 5-, and 6-day old larval gut samples (Am4CK, Am5CK, Am6CK groups) were prepared and subjected to RNA extraction, cDNA library construction, and deep sequencing using the Illumina MiSeq platform by Genedenovo Biotechnology Co., Ltd. (Guangzhou, China), followed by the strict quality control of raw data [47,48,49]. Three larval guts were mixed as a group, and each group included three biological replicas. The high-quality raw data produced from sRNA-seq were deposited in the NCBI Sequence Read Archive (SRA) database (http://www.ncbi.nlm.nih.gov/sra/, accessed on 18 September 2020) under the BioProject number: PRJNA565629.

### 4.3. Bioinformatic Prediction and Analysis of piRNAs

piRNAs were predicted according to our previously established procedure [16]. Briefly, (1) the clean reads were mapped to the *A. mellifera* genome (assembly Amel_4.5) to obtain mapped reads; (2) the mapped clean tags were aligned to the GenBank [50] and Rfam [51] databases using the blastn tool to remove rRNA, scRNA, snoRNA, snRNA, and tRNA; (3) miRNAs were filtered from the remaining clean reads; (4) sRNAs with a length distribution between 24 nt and 33 nt were screened on the basis of the length characteristics of piRNAs, and only those aligned to a unique position were retained as candidate piRNAs; (5) candidate piRNAs were further compared to piRBase [52] using bowtie software (http://ftp://ftp.ccb.jhu.edu/pub/data/bowtie_indexes/, accessed on 18 September 2020) to obtain piRNAs present in the database.

The expression level of each piRNA was normalized and calculated according to TPM = T × 10^6^/N (T denotes clean reads of piRNA; N denotes clean reads of total sRNA). Next, the length distribution and first base bias of piRNAs were investigated based on the prediction result. The UpSet plot and Ridgeline plots of the piRNA expression levels were visualized using the OmicShare platform (https://www.omicshare.com/tools/, accessed on 5 December 2022). The parameters were set as: statistical threshold > 0 in the upset plot, Zoom = 1, and Bandwidth = 1.

### 4.4. Identification of DEpiRNAs

The fold change of the expression level of each piRNA between different groups was determined following the formula: (TPM in Am4CK)/(TPM in Am4T) and (TPM in Am5CK)/(TPM in Am5T) or (TPM in Am6CK)/(TPM in Am6T). Based on the criteria of |log_2_FC| ≥ 1 and *p* ≤ 0.05, DEpiRNAs in the Am4CK vs. Am4T, Am5CK vs. Am5T, and Am6CK vs. Am6T comparison groups were screened using edgeR software (http://www.bioconductor.org/ version 4.2, accessed on 27 October 2022) [53]. A Venn analysis of DEpiRNAs in each comparison group was conducted using the related tool in the OmicShare platform (https://www.omicshare.com/tools/Home/Soft/venn, accessed on 27 October 2022) with default parameters.

### 4.5. Prediction and Investigation of DEpiRNA-Targeted mRNAs

Targets prediction was performed by blast [54], the piRNA sequences were compared to the genomic sequences using blastn software (version 2.2.25), and only those that were precisely matched and complementary to each other were retained (three mismatches were allowed). Then, each sequence that could be targeted by the piRNAs was scored to predict the piRNA target loci and target mRNAs, the targets of piRNA expressed differently among different samples were analyzed statistically, and the Union set was used for enrichment analysis. Next, the targets were respectively annotated in the GO (https://www.geneontology.org (accessed on 6 December 2021)) and KEGG (https://www.genome.jp/kegg/ (accessed on 2 November 2023)) databases to gain corresponding functional and pathway annotations.

### 4.6. Analysis of the DEpiRNA-mRNA Regulatory Network

Based on the predicted targeting relationships, regulatory networks between DEpiRNA and target mRNAs were constructed following the thresholds of free energy < −10 kcal mol ^−1^. Further, on the basis of the KEGG pathway annotations, the target mRNAs annotated in Endocytosis, ubiquitin-mediated proteolysis, the metabolism of xenobiotics by cytochrome P450 associated with immune-related pathways; FoxO, the Wnt signaling pathway, and the Hippo signaling pathway-fly were associated with development-related signaling pathways; oxidative phosphorylation, nitrogen metabolism, and sulfur metabolism associated with energy metabolism-related pathways were further surveyed to construct corresponding regulatory networks, which were then visualized with the Cytoscape software (Version: 3.3.0) [55].

### 4.7. Validation of DEpiRNAs by Stem-Loop RT-PCR

The total RNA from the gut tissues of 4-, 5-, and 6-day-old larvae were isolated with the FastPure Cell/Tissue Total RNA Isolation Kit v2 (Vazyme, Nanjing, China), followed by an evaluation of the purity and concentration using a Nanodrop 2000 spectrophotometer (Thermo Fisher, Waltham, MA, USA). One DEpiRNA (piR-ame-945760) in the Am4CK vs. Am4T comparison group, two (piR-ame-1186994, piR-ame-904316) in the Am5CK vs. Am5T comparison group, and two (piR-ame-978292, piR-ame-1199278) in the Am6CK vs. Am6T comparison group were randomly selected for RT-PCR validation. Specific stem-loop primers, as well as upstream primers (F) and universal downstream primers (R), were designed using DNAMAN software (Version:8.0.8.789) and then synthesized by Sangon Biotech Co., Ltd. (Shanghai, China). According to the instructions of the HiScript^®^ 1st Strand cDNA Synthesis Kit (Yeasen, Shanghai, China), reverse transcription was performed with stem-loop primers, and the obtained cDNAs were used as templates for the PCR of DEpiRNA, Reverse transcription was performed using a mixture of random primers and oligo (dT) primers, and the resulting cDNA was used as templates for the PCR of the reference gene snRNA *U*6 (GenBank ID: LOC725641), which was conducted on a T100 thermocycler (Bio-Rad, Hercules, CA, USA) under the following conditions: pre-denaturation at 95 °C for 5 min, 40 amplification cycles of denaturation at 95 °C for 10 s, annealing at 55 °C for 30 s, and elongation at 72 °C for 1 min, followed by a final elongation step at 72 °C for 10 min. The PCR system (20 μL) included 1 μL of diluted cDNA, 10 μL of PCR mix (Yeasen, Shanghai, China), 1 μL of forward primers, 1 μL of reverse primers, and 7 μL of DEPC water. The amplification products were checked on 1.8% agarose gel electrophoresis with Genecolor (Gene-Bio, Shenzhen, China) staining.

### 4.8. RT-qPCR Verification of DEpiRNAs and Target mRNAs

The above-mentioned five randomly selected DEpiRNAs were carried out following the protocol of the SYBR Green Dye kit (Vazyme, Nanjing, China), which was conducted on an Applied Biosystems QuantStudio 3 system (Thermo Fisher, Waltham, MA, USA) with the following conditions: pre-denaturation step at 95 °C for 5 min, 40 amplification cycles of denaturation at 95 °C for 10 s, annealing at 60 °C for 30 s, and elongation at 72 °C for 15 s, followed by a final elongation step at 72 °C for 10 min. The reaction system included 1.3 μL of cDNA, 1 μL of forward primers, 1 μL of reverse primers, 6.7 μL of DEPC water, and 10 μL of SYBR Green Dye. All reactions were performed in triplicate. The relative expression level of each DEpiRNA was calculated following the 2^−ΔΔCt^ method [56]. Detailed information about the primers used in this work is presented in Table S1.

Based on the target prediction and annotation results, six target mRNAs (sorting nexin-6 (*SNX*6, GenBank ID: NC_037638.1); suppressor of cytokine signaling 5 (*SOCS*5, GenBank ID:NC_037638.1); V-type proton ATPase subunit d (*ATP*6*V*1*D*, GenBank ID: NC_037643.1); E3 ubiquitin-protein ligase TRIM37-like (*E*3*UBL-TRIM*37, GenBank ID: NC_037640.1); ubiquitin-conjugating enzyme E2 Q2 (*UBE E*2 *Q*2, GenBank ID: NC_037638.1); AP-1 complex subunit gamma-1 (*AP*1*G*1, GenBank ID: NC_037649.1)) associated with five immune pathways—endocytosis, the Jak-STAT signaling pathway, oxidative phosphorylation, ubiquitin-mediated proteolysis, and lysosome—were selected for RT-qPCR determination. Reverse transcription was performed using a mixture of random primers and oligo (dT) primers, and the resulting cDNA was used as templates for the PCR of the six targets and the reference gene *actin* (GenBank ID: NC_037644.1). According to the instructions of the SYBR Green Dye kit (Vazyme, Nanjing, China), the reactions were performed on an ABI QuantStudio 3 fluorescence quantitative PCR instrument with the following conditions: pre-denaturation at 95 °C for 5 min, denaturation at 95 °C for 10 s, and annealing and extension at 60 °C for 30 s for 40 cycles. The reaction system contained 10 μL of SYBR Green Dye, 1.3 μL of the cDNA template, 1 μL of forward and reverse primers, and 6.7 μL of DEPC water. All reactions were performed in triplicate. The relative expression level of each DEpiRNA was calculated using the 2^−ΔΔCt^ method. Detailed information about the primers used in this work is presented in Table S1.

### 4.9. Overexpression and Knockdown of piR-ame-945760 in A. apis-Infected Larval Guts

According to the method described by Wu et al. [44], piR-ame-945760 mimic (mimic-piR-945760) and inhibitor (inhibitor-piR-945760), as well as corresponding negative controls (mimic-NC and inhibitor-NC), were designed using Primer Designer software (Version: 1.30) and synthesized by GenePharma (Shanghai, China) (Table S2). *A. m. ligustica* larvae were reared following the methods developed by Peng et al. [57], with minor modifications. In brief, (1) the queen in an *A. m. ligustica* colony was confined to the empty comb using the queen spawning controller, and after 15 h of natural oviposition, the queen was taken out and the comb was quickly transferred to the laboratory; (2) 2-day-old larvae were carefully transferred to six-well culture plates (each well was preset with 800 μL of an artificial diet), with 40 larvae per well, and the plates were then placed in a constant temperature and humidity incubator (35 ± 0.5 °C, RH 90%) for 24 h; 3-day-old larvae were transferred to 48-well culture plates, and each larva was fed 5 μL of *A. apis* spores at a final concentration of 4 × 10^5^/mL at 10:00 a.m. and then fed 45 μL of a diet containing mimic-piR-945760, mimic-NC, inhibitor-piR-945760, or inhibitor-NC (40 pmol/g). These four groups were respectively named: (1) *A. apis* + mimic-piR-945760 group, (2) *A. apis* + mimic-NC group, (3) *A. apis* + inhibitor-piR-945760 group, and (4) *A. apis* + inhibitor-NC group. After that, 45 μL of the diet was added to each well per 12 h. Then using clean ophthalmic scissors and tweezers, the 4-, 5-, and 6-day-old larval guts were dissected, the fat bodies that adhered to the guts were carefully removed, and every three larval gut samples were placed in one sterile Rnase-free Eppendorf tube. After being frozen with liquid nitrogen, the gut samples were transferred to a −80 °C refrigerator immediately for cryopreservation. There were three biological replicas for this experiment.

Following the method mentioned above, the gut samples were subjected to RNA isolation and cDNA synthesis followed by RT-qPCR detection to evaluate the effects of piR-ame-945760 overexpression and knockdown in the *A. apis*-infected larval guts.

### 4.10. RT-qPCR Detection of piR-ame-945760-Targeted mRNAs

Based on the target prediction result, two target mRNAs of piR-ame-945760 were selected for RT-qPCR detection, including the suppressor of cytokine signaling 5 gene (*SOCS*5, GenBank ID: NC_037638.1) and the ADP-ribosylation factor 1 gene (*ARF*1, GenBank ID: NC_037638.1). The forward and reverse primers for both *SOCS*5 and *ARF*1 (Table S1) were designed and synthesized by Sangon Biotech Co., Ltd. (Shanghai, China). According to the aforementioned method, the total RNA in 4-day-old *A. apis*-infected larval guts was isolated, and reverse transcription was then performed using a mixture of random primers and oligo (dT) primers, followed by RT-qPCR with the obtained cDNA as templates. The *actin* gene (GenBank ID: NC_037644.1) was selected as the inner reference. All reactions were performed in triplicate. The relative expression level of each target gene was calculated using the 2^−ΔΔCt^ method.

### 4.11. Statistical Analysis

Statistical analyses were conducted with SPSS 26.0 software (IBM, Amunque, NY, USA) and GraphPad Prism 7.0 software (GraphPad, San Diego, CA, USA). Data are shown as the mean *±* standard deviation (SD) and were subjected to Student’s *t*-test. Fisher’s exact test was performed using R software 3.3.1 to screen significant (*p* < 0.05) GO terms and KEGG pathways.

## 5. Conclusions

**Figure 12:**
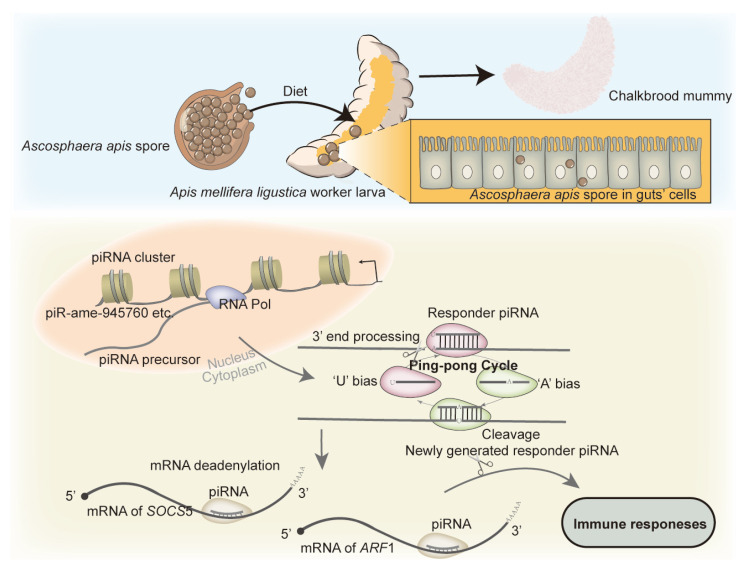
A hypothetical working model of the DEpiRNA-modulated responses of *A. m. ligustica* worker larvae to *A. apis* invasion. On the basis of the results obtained in this study, differentially expressed piRNAs (DEpiRNAs) were potential modulators in the larval response to *A. apis* invasion by targeting and regulating the expression of mRNAs of host genes like *SOC*5 and *ARF*1. At the bottom of the diagram, the brown curve within a mRNA represents 5’ UTR, while the orange curve within a mRNA represents CDS region.

In summary, a total of 772 piRNAs were identified in the gut of *A. m. ligustica* larvae infected by *A. apis*, with the length distribution and first base bias similar to those of piRNAs in mammals; the *A. apis* infection increased the overall expression level of piRNAs and changed the expression pattern of piRNAs in larval guts; DEpiRNAs were putative regulators in the *A. apis*-response of *A. m. ligustica* worker larvae; and piR-ame-945760 negatively regulated the expression of *SOCS*5 and *ARF*1 in the larval guts during the *A. apis* infection (Figure 12).

## Data Availability

Data are contained within the article and Supplementary Materials.

## Supplementary Materials

The following supporting information can be downloaded at: https://www.mdpi.com/article/10.3390/ijms242216358/s1.
