# Supplementary material for: First Characterization and Regulatory Function of piRNAs in the Apis mellifera Larval Response to Ascosphaera apis Invasion
Source: Int J Mol Sci. 2023 Nov 15;24(22):16358. doi: 10.3390/ijms242216358 (PMC10671575; doi:10.3390/ijms242216358)
Supplement: Supplementary file 1 [file ijms-24-16358-s001.zip › Figure S1.pdf]

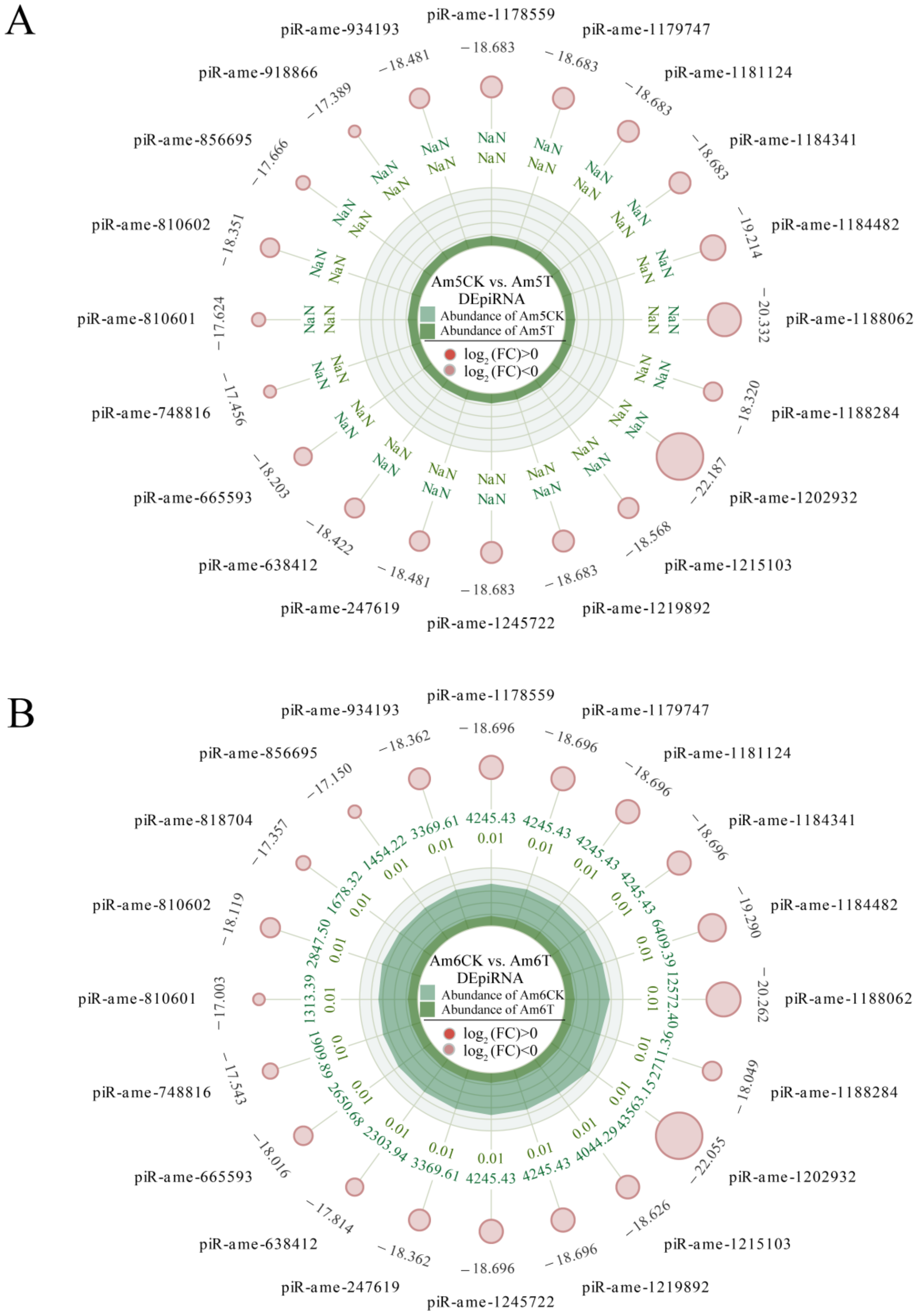

Figure S1. Radar map of the top 20 DEpiRNAs in the Am5CK vs. Am5T (**A**) and Am6CK vs. Am6T (**B**) comparison group, green circles indicate down-regulated piRNAs, and the size of each circle indicates the numerical value of $\log_2$(FC); data outside the circles indicate the average expression of piRNAs in the Am5CK or Am6CK group; and inner-circle data represent the average expression of sample Am5T or Am6T; the light and deep green colors at the center repectively indicate the expression abundance of

Am5CK or Am6CK and Am5T or Am6T on each axis.
