# Supplementary material for: First Characterization and Regulatory Function of piRNAs in the Apis mellifera Larval Response to Ascosphaera apis Invasion
Source: Int J Mol Sci. 2023 Nov 15;24(22):16358. doi: 10.3390/ijms242216358 (PMC10671575; doi:10.3390/ijms242216358)
Supplement: Supplementary file 1 [file ijms-24-16358-s001.zip › Figure S2.pdf]

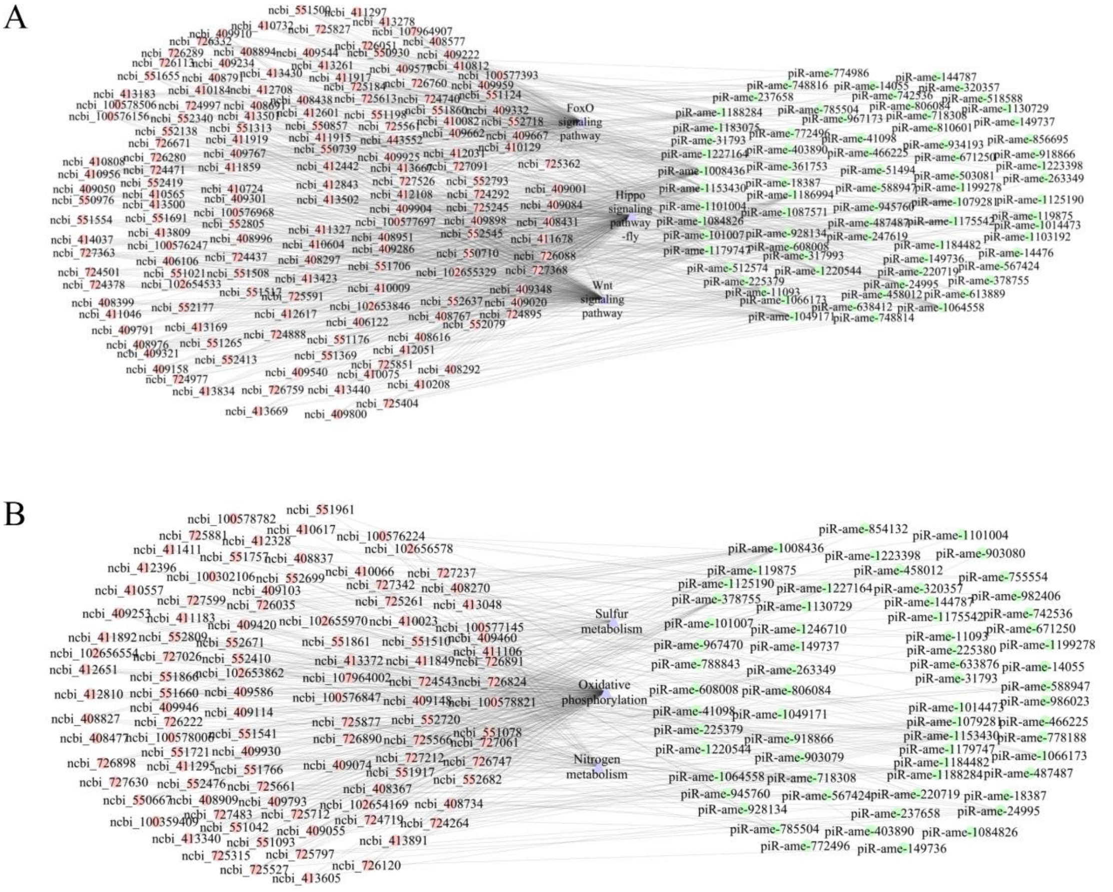

Figure S2. Development pathway-associated regulatory network (**A**) and energy metabolism pathway-associated regulatory network (**B**) between DEpiRNAs and target mRNAs in the 4-, 5-, and 6-day-old comparison groups. Pink and green circles respectively indicate target mRNAs and DEpiRNAs, while purple triangles indicate immune pathways.
